# An Improved Approach to Direct Simulation of an Actual Almen Shot Peening Intensity Test with a Large Number of Shots

**DOI:** 10.3390/ma13225088

**Published:** 2020-11-11

**Authors:** Chengyu Wang, Weigang Li, Jianjun Jiang, Xin Chao, Weikui Zeng, Jiang Xu, Jie Yang

**Affiliations:** 1 School of Mechanics and Engineering, Southwest Jiaotong University, Chengdu 610031, China; Chengyuwang@my.swjtu.edu.cn (C.W.); atfangi030@163.com (W.L.); 2 Engineering Department, Chengdu Aircraft Industry (Group) Co. LTD., Chengdu 610091, China; j3why@aliyun.com (J.J.); chaoxin_132@126.com (X.C.); scuzwk@163.com (W.Z.); 3 School of Aeronautics, Northwestern Polytechnical University, Xi’an 710072, China

**Keywords:** shot peening, almen intensity, improved DEM–FEM, transverse deformation, surface plastic layer, precision peen forming

## Abstract

In existing simulations of the Almen intensity test, arc height is indirectly obtained by an equivalent method including a representative cell, a few shots and equivalent loading. Most of these equivalent methods cannot consider the transverse deformation of the strip, the complex stress state of the plastic hardening layer and process parameters, resulting in deviation from the actual test. This paper introduces an improved and experimentally validated discrete element model (DEM)-finite element model (FEM) to predict the actual Almen intensity. The improvement of this model is mainly reflected in the large and real number of shots involved in the actual Almen intensity test, shot–shot interactions, and real-size solid finite element model of the Almen strip. A new method for calculating the shot stream is proposed based on the test and considering test process parameters such as the mass flowrate, nozzle movement speed and nozzle–workpiece distance. The shot stream impacting the strip with a fully restrained underside was first simulated in improved DEM-FEM to bring the forming energy. As a second step, an implicit solver of the Almen strip FEM calculates the spring-back to simulate strip removal from the holder. The results achieved by the present approach are compared with the results obtained by the experimental results and those in the literature. The results show that the arc height and Almen intensity obtained by the present approach match much better with the literature than the traditional method. Some new results obtained by the improved coupling DEM-FEM method are presented. The influences of the transverse deformation and surface plastic layer on the deformation of the Almen strip are discussed. This improved method provides an alternative characterization method for precision peen forming simulation.

## 1. Introduction

The shot peening process is employed as a mature industrial cold processing method and is widely used in the aerospace and automation fields. In this process, numerous tiny high-velocity shots impact the treated surface of the component to cause plastic deformation of the surface layer. Thus, compressive stresses are introduced near the surface. The fatigue life of the part is dramatically improved under the compressive residual stress effect [1]. To balance the compressive stresses near the surface, the outer plastic layer must be stretched. Therefore, this process is also frequently used in the forming of thin parts to shape a desired curvature or to correct the shape of a component, especially for forming complex contoured aircraft skin panels [2]. The process is effective in producing significant shape deformation by increasing the fatigue resistance of the surface [3].

In actual shot peening, the Almen intensity test is a common process control variable in peening applications since it represents the combined effect of all parameters simultaneously and at relatively low cost. Bhuvaraghan and Srinivasan [4] characterized the arc height under different exposure times, which can be measured by the Almen gauge. The Almen intensity is defined as the arc height of the Almen strip at saturation point [5]. Miao and Demers [6] reported that quantitative relationships between the saturation, coverage and surface roughness regarding exposure time have been obtained based on Almen-sized Al2024 strips. Kumar and Sampath Kumaran [7] designed experiments and established a relationship between the Almen intensity and air pressure based on titanium alloy. However, the Almen strip test has certain limitations. For instance, Bhuvaraghan and Srinivasan [4] noted that the same intensity can be produced with distinct combinations of shot sizes and velocities, which can cause different residual compressive stress patterns. In addition, Gariépy and Larose [5] noted that the Almen intensity test fails to provide detailed information on the material state during and after peening, especially for materials other than SAE 1070 steel.

Some scholars also studied the Almen intensity by theoretical analysis. For example, Miao and Larose [8] proposed a model for predicting the Almen intensity based on an analytical model for shot peening residual stresses with different peening parameters. Zhang and Wang [9] suggested that an analytical model of the shot peen forming process can be established by using the cross-sectional linear indentation coverage method. Sherafatnia and Farrahi [10] related the elastoplastic unloading of shot impingements, friction coefficient effect and fraction of kinetic energy transmitted to the treated material. The limitation of the theoretical analysis method is that the details of the shot peening process cannot be reflected.

With the development of numerical methods, many scholars have established finite element models (FEMs) to simulate Almen intensity test. Simulation methods of the shot peening process have developed from 2D models to 3D models, from shot deterministic positions to shot random positions. In all simulations, a representative elementary volume—i.e., a 3D unit cell from the treated structure—is adopted to obtain residual stress and surface roughness. The results of the representative elementary volume method show that the numerical simulation can accurately predict the shot peening effect in terms of the residual stress and coverage. However, this representative elementary volume method cannot directly calculate the deformation of the Almen strip. Generally, in the current study, deformation simulation of the Almen strip is divided into two steps. In the first step, induced stress is obtained by a small number of shots repeated impacting representative elements [11]. For example, Lin and Liu [12] and Sheng and Xia [13] established a random multiple shots model and 3D small volumes to explore the effects of different shot velocities and shot numbers on the residual stress of shot peening. However, shot peening of a small structural volume in the vicinity of geometrical details, such as notches and sharp edges, could not be simulated by a small number of shots [12]. In the current study, most researchers used the FEM method to obtain the residual stress and surface roughness, and other researchers combined the discrete element model (DEM) and FEM to reflect a large number of shots distribution and real coverage. For example, Tu and Delbergue [13], Murugaratnam and Utili [14] and Jebahi and Gakwaya [15] applied a combination of DEM and FEM to study the residual stress and other effects by rigid shots and the representative elementary volume method. In the second step, the unbalanced induced stress was used to calculate the arc height by a theoretical model or FE model using an equivalent approach. For example, Guagliano [16] calculated the arc heights of Almen strips from the bending moment equilibrating the residual stress field and arc height (*A_h_*) given by Equations (1) and (2):

(1) $$M_{x}=-\int_{0}^{k}\sigma_{x}\left(\frac{k}{2}-z\right)wdz$$

(2) $$A_{h}=\frac{3M_{x}L^{2}}{2Ewk^{3}}$$

where *E* is the strip’s Young’s modulus, and *L*, *W* and *k* are the strip length, width and thickness, respectively. In most existing numerical methods, the arc height of the Almen strip is obtained by applying induced stress layer by layer on the Almen strip shell model. The methods mainly include the thermal loading equivalent method and the induced stresses equivalent method to simulate the peen forming process. For example, Han [17] proposed an explicit/implicit simulation strategy to model peen forming based on residual stress. Wang and Platts [18] presented the equivalent thermal loading model by using shell elements to obtain the deformation of the strip. Gariépy and Cyr [19] and Miao and Larose [20] used a shell element-based model and defined in-plane stress values as inputs at section points. There are other numerical methods. Bhuvaraghan and Srinivasan [4] used an FEM technique to model a slice of the Almen strip and captured its response behavior directly. Edward and Heyns [21] reported a simplified methodology for the simulation of the shot peening process and replaced the transient analysis with a simpler static structural analysis. Even though the existing numerical methods of Almen intensity can greatly reduce the cost and obtain more details that are difficult to find during the experiment, these simulation methods cannot directly simulate the Almen intensity, which is different from the real situation. 

The motivation of this paper is to replicate the actual Almen intensity test by numerical methods method as much as possible. Direct simulation of the actual Almen intensity test by a large and real number of random shots based on improved DEM–FEM is presented in this paper. To our knowledge, this paper is the first to simulate a large and real number of shots impacting the Almen intensity strip and obtain the deformation directly. The advantages of this approach are that the improved DEM–FEM method is designed based on the Almen intensity experiment, the shot model reflects the various process parameters of actual shot peening, and the strip size in the simulation is a true size rather than a representative unit. A new method for calculating the number of shots based on the shot peening test considering various peening parameters is presented in this paper. The influence of the transverse deformation and surface plastic layer on the deformation of the Almen strip was studied, and the predicted Almen intensity at different air pressures was compared to experimentally measured results and the literature.

The paper is organized as follows. Section 2 introduces the equipment and materials in the shot peening process used for generating the experimental results. A new method for calculating the shot stream number needed for simulation is proposed. Section 3 addresses the improved discrete element model (DEM)-finite element model (FEM) and the method for directly obtaining the arc height corresponding to the Almen intensity test. Section 4 shows the experimental data, and the simulation results for arc height and Almen intensity and the method in this paper are fully verified. Comparison study of the improved DEM-FEM method and other existing methods is discussed in Section 5. Finally, conclusions are presented in Section 6.

## 2. Experimental Procedure

### 2.1. Shot Peening Experiment 

**Figure 1:**
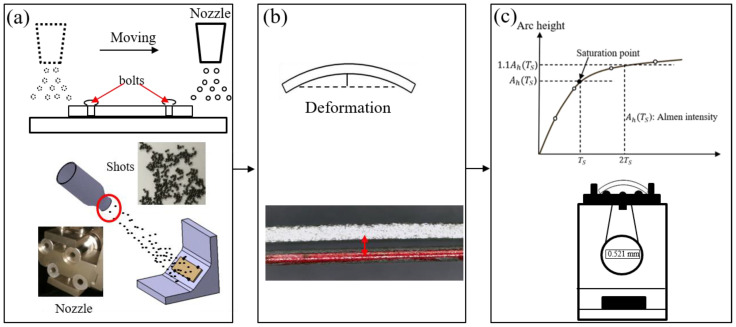
Almen intensity test process. (**a**) The strips are fixed on their holders. (**b**) After a certain amount of coverage, the strips are removed from the holders. The strips bend and extend. (**c**) This arc height is measured by the Almen gauge and Almen intensity is obtained by saturation curve.

**Figure 2:**
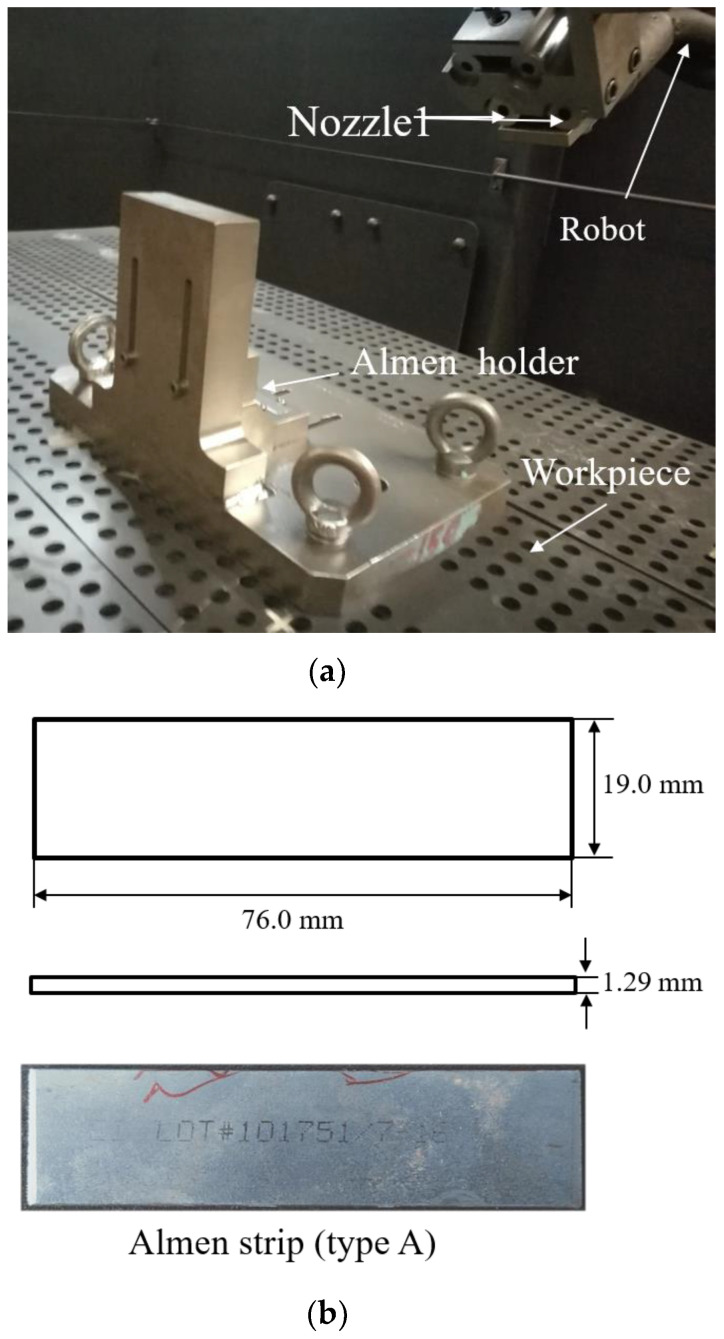
Equipment. (**a**) Shot peening equipment. (**b**) An Almen strip of type A.

Figure 1 shows the shot peening process of Almen intensity test. The test consists of peening a standardized Almen strip of given dimensions and material that is clamped to a mounting fixture with four roundhead pretension bolts. The shot peening experiment is accomplished with a high-precision mobile blasting machine. The blasting machine and the nozzle (with an 8 mm diameter at a working distance of 300 mm) is shown in Figure 2a. A device called the Almen holder is used to fix the strip to the metal block with four bolts. Strips are mounted on their holders and subjected to the same peening conditions as the actual parts. In the actual shot peening process, Almen intensity is a simple and rapid standard method for evaluating shot peening effects, and is controlled by air pressure and other parameters instead of shot velocity. In this paper, the moving velocity of the nozzle *V_R_* was 1000.0 mm/min, and the mass flowrate was 6.0 kg/min. S230 stainless steel shots with a diameter of 0.58 mm were used in all experiments. The operation parameters were selected for air pressure, *P*, as 1.0, 1.5, 2.0, and 2.5 bar. An Almen strip of type A was selected as the research object, as schematically displayed in Figure 2b. At least four Almen strips per group were used to determine the saturation curves for certain air pressures. The peening time increment can be defined by considering different numbers of peening passes at a consistent robot traveling velocity of 1000.0 mm/s.

### 2.2. Measuring Arc Height

**Figure 3:**
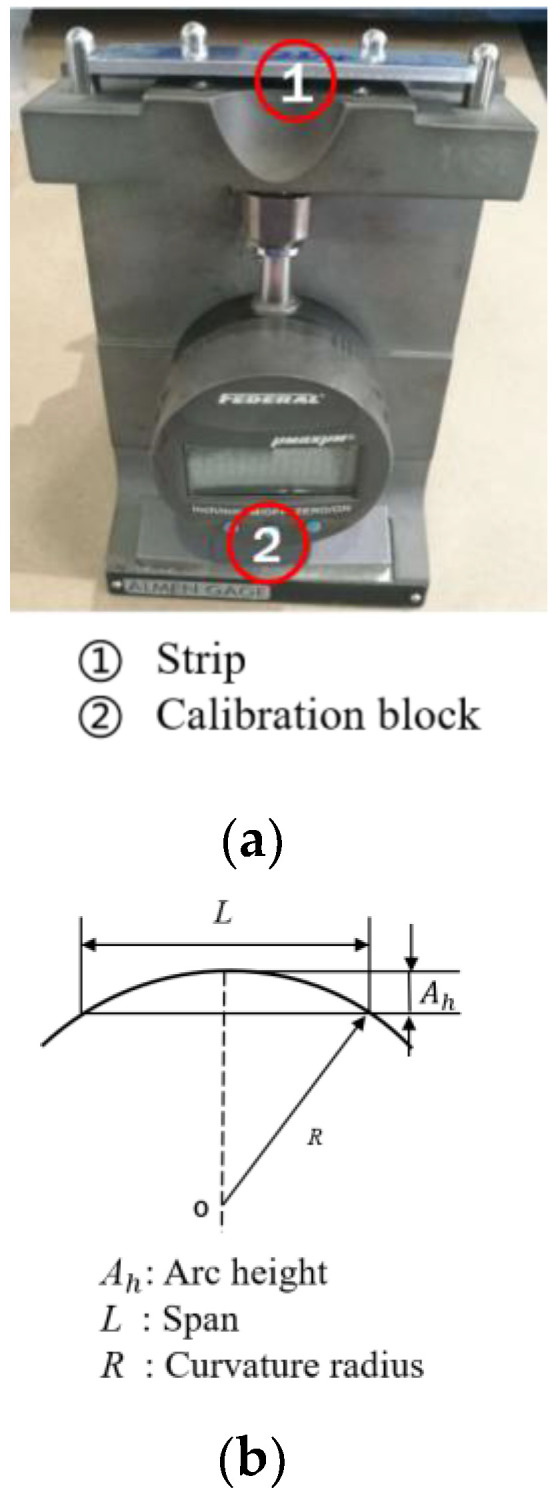
Measuring the arc heights of Almen strips. (**a**) Almen gauge. (**b**) Arc height measurement schematic.

The total arc heights include two parts: deformation along the longitudinal direction, length 31.75 mm, and along the transverse direction, width 15.87 mm [6]. The relationship between the curvature radius *R* and the arc height *A_h_* can be obtained by a geometrical analysis, as schematically displayed in Figure 3, which is expressed by Equation (3):

(3) $$A_{h}=\frac{L^{2}}{8R}$$

where *L* is the length of the span (Hu and Zhang [22]).

## 3. The Improved DEM–FEM Approach 

### 3.1. Overall Strategy

**Figure 4:**
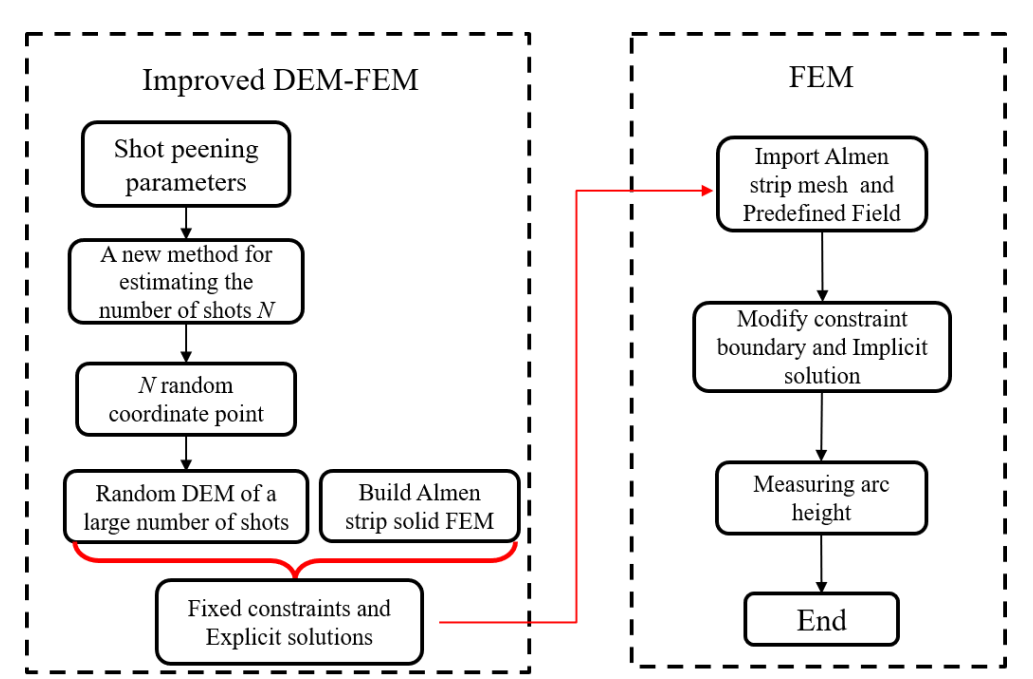
The proposed improved discrete element model (DEM)-finite element model (FEM) scheme of a large number of random shots, which simulates the actual Almen intensity test. The shot stream number is calculated by a new method based on the test and shot peening parameters. The random coordinate point of the shot stream is obtained by Python program. A large and real number of random shots hitting the Almen strip is established in Abaqus/Explicit. The extracted Almen strip mesh and predefined field are imported into Abaqus/Implicit to obtain the spring back.

The proposed DEM-FEM coupling method of a large and real number of shots is an improvement of the method introduced by Jebahi and Gakwaya [15], Edward and Heyns [21] and Zhang and Lu [23]. The advantage of the improved method lies in the fact that the shot peening process is sequentially simulated without the need to extract the average equivalent induced stress to obtain the deformation indirectly. First, the shot stream number is calculated by a new method based on the test and shot peening parameters. Second, the random coordinate point of the shot stream is obtained. Third, a large and real number of random shots hitting the Almen strip with a fully fixed underside is established in Abaqus/Explicit (6.16). Finally, the extracted Almen strip mesh and predefined field are imported into Abaqus/Implicit to simulate the spring back by modifying constraint boundary. A simplified workflow of the improved approach is shown in Figure 4.

**Figure 5:**
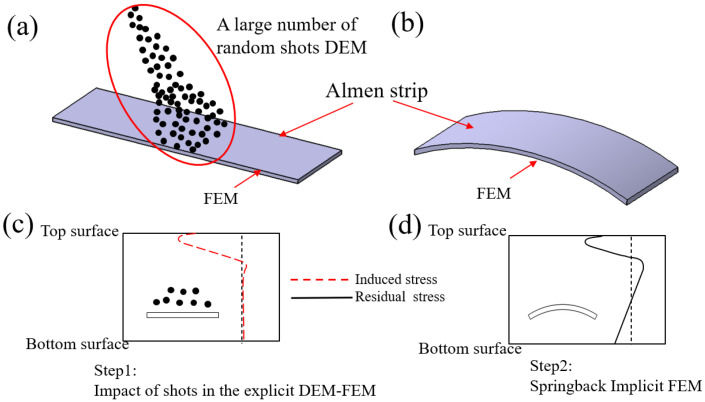
Improved DEM-FEM method. (**a**) Impact of shots in the explicit DEM-FEM. (**b**) Springback Implicit FEM. (**c**) Induced stress profile. (**d**) Residual stress profile.

The improved DEM–FEM coupling method is used to simulate major mechanical phenomena of shot peening, as shown in Figure 5. In short, in the first step, an explicit solver calculation based on improved DEM–FEM is used to execute a large and real number of random shots impacting the restrained strip, which depicts the real situation of impacting the strip mounted on their holders and, therefore, to bring the forming energy to bear on the strip as shown in Figure 5a. In the second step, implicit solver calculations based on FEM are performed to simulate strip removal from the holder though the technical release of constraints. Through vast impacts on the Almen strip, which is mounted into place using the Almen holder, the energy stored in the vast shots transfers into the peened material and produces a compressive residual stress layer near the surface, as shown in Figure 5c. After the strips are removed from the holders, and due to the change in the boundary conditions, the nonequilibrated stress forces the Almen strip to bend toward the peening direction to form the arc height, as shown in Figure 5d.

### 3.2. Material Model

The peening simulation is applied on an Almen strip of type A. S230 stainless steel shot is used in all calculations. The target component is modeled via FEM. Since very high strain rates are involved in the shot peening process, rate-dependent properties must be considered in the target component model. There are many well-verified mathematical models that consider strain rates in evaluating the stress–strain relations (Huang, Wang [24]). Based on the work of Jebahi and Gakwaya [15], the isotropic hardening approach with rate-dependent properties correctly predicts the mechanical response of an Almen strip undergoing shot peening. Therefore, this Johnson–Cook constitutive model is applied in this study. Here, the Johnson–Cook equation is employed to evaluate the stress–plastic strain relations:

(4) $$\sigma =\left[A+B\varepsilon^{n}\right]\left[1+C\ln\left(\frac{\varepsilon }{{\dot{\varepsilon }}_{0}}\right)\right]\left[1-{\left(\frac{T-T_{0}}{T_{m}-T}\right)}^{m}\right]$$

where σ is the stress to be evaluated; ${\dot{\varepsilon }}_{0}$, *T*_0_ are the reference values of strain rate and temperature, respectively; $\dot{\varepsilon }$, *T* are the strain rate and temperature under consideration, respectively; *Tm* is the melting temperature; and *A*, *B*, *C*, *n* and *m* are the five Johnson–Cook constants to be determined experimentally. In the current study, the effect of temperature increase due to peening is not included. Therefore, only the constants *A*, *B*, *C* and *n* are evaluated for the materials. In this work, the mechanical properties, and the Johnson–Cook constants of the shot and Almen strip are given in Table 1 (Jebahi and Gakwaya [15]).

### 3.3. A New Method for Calculating the Shot Stream Number

**Figure 6:**
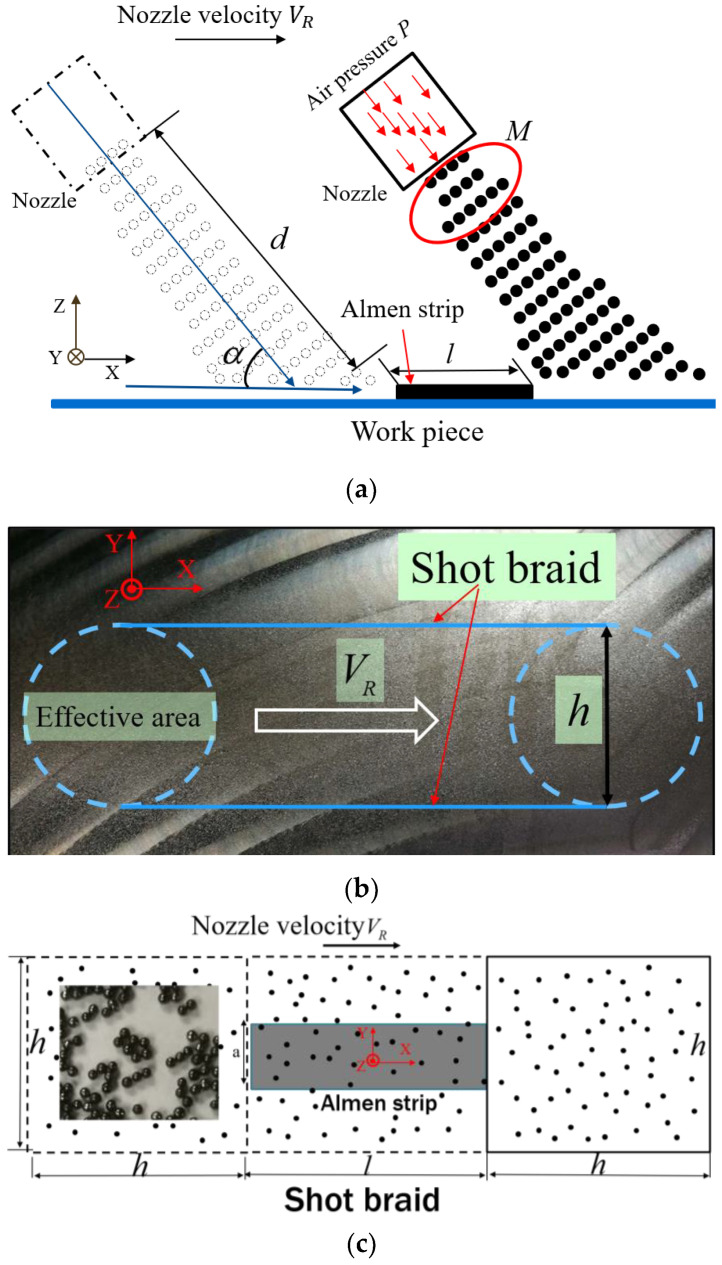
The proposed calculation method for the number of shots. (**a**) Peening parameters for calculating the number of shots. (**b**) The effective square area on the plate. (**c**) Simplified schematic diagram for calculating the number of effective shots impinged on the Almen strip.

Determining the number of shots involved in the Almen intensity test is the first step in the simulation process. Figure 6a illustrates that the shot distribution is related to the impact angle; the width of the shot braid is dependent on the distance from the nozzle to the workpiece; and the coverage rate is determined by the shot velocity, impact angle and mass flowrate. In short, the shot braid is influenced by many shot peening process parameters. These shot peening parameters can all be considered for calculating the shot number as proposed in this paper. This paper introduces a new method to calculate the number of shots used in simulation, considering the mass flow and other parameters of shot peening. The indentations are distributed in a zonal pattern along the track of the nozzle and normally distributed in the direction perpendicular to the track. To simplify the problem, we assume that the effective area of the indentations is a square and that indentations in the square are distributed randomly and uniformly as shown in Figure 6b. In the actual shot peening, not all shots hit the Almen strip because of the relatively small size of the strip. The simplified schematic diagram for calculating the number of effective shots impinged on the Almen strip is presented in Figure 6c. *N_T_* is the number of total shots in the experiment. *N* is the number of effective shots needed in the simulation. *t* is the effective time for the shot impacting the strip with the nozzle moving at a speed of *V_R_*, and the effective distance traveled is *l* + *h*. The definitions of *N_T_* and *N* are presented in Equations (6) and (7), respectively.

(5) $$t=\frac{\left(l+h\right)}{V_{R}}$$

(6) $$N_{T}=\frac{Mt}{m_{1}}$$

(7) $$N=\frac{A}{h(l+h)}N_{T}$$

where *m*_1_ is the mass of a single shot, *l* is the effective distance over which the shots impact the surface of the part, *h* is the edge length of the effective shot peening area, *A* is the area of the strip, *V_R_* is the velocity of the nozzle, and *M* is the mass flowrate.

### 3.4. Improved DEM–FEM Approach

#### 3.4.1. The First Model: Improved DEM–FEM for Impacting 

During a shot peening process, a large number of shots impact the treated component at random positions and in a random sequence. In the present work, the number of shots is controlled by the nozzle movement velocity for a given shot type, mass flow, distance from the nozzle to the strip and impact angle. The shot number for simulation is calculated by using Equation (7). In this paper, it is assumed that *h* is 70 mm, corresponding to the distance from the nozzle to the strip; *l* is 76 mm; and *V_R_* is 1000.0 mm/min. The number of shots in the simulation is shown in Table 2. 

**Figure 7:**
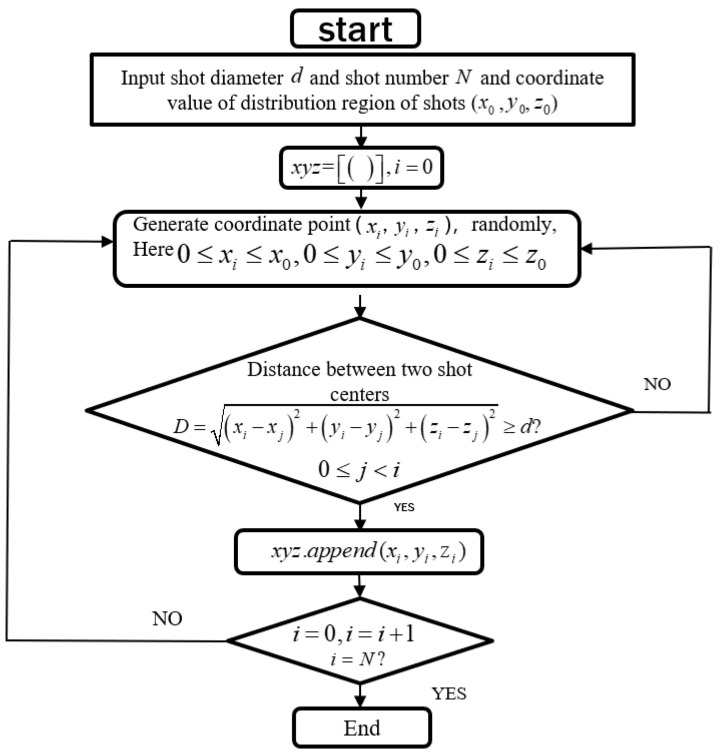
Python program frame for random coordinates of *N* shots.

In an improved DEM-FEM impacting simulation, these discrete elements (PD3D) of a random shot are simulated only by their density *ρ*, radius *R* and coordinates of their centers *x, y, z*. To randomly generate the distributed shots, a python program (3.7.2) is combined with Abaqus/Explicit. This program requires as inputs the Almen strip peening space (*x × y × z*), shot number N and radius R. The z-axis is normal to this peening surface and directed towards the Almen strip. Assuming an (*x × y × z*) space, in the case of normal impacts, the coordinates of each shot center are randomly generated according to the Python program frame (Figure 7).

**Figure 8:**
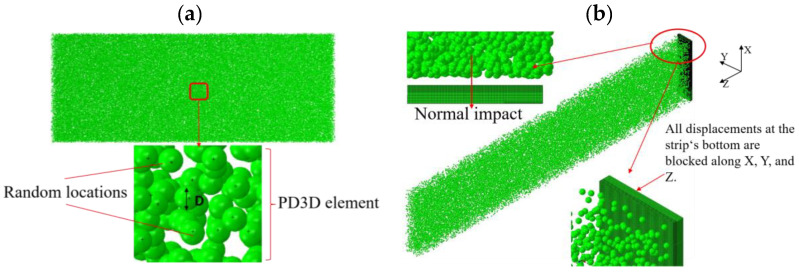
DEM–FEM for shot peening. (**a**) Three-dimensional random shot model. (**b**) Normal impact of the shots in the explicit DEM–FEM model.

For the sake of simplicity, the initial velocity is applied to all the shots in the impingement direction. The nonlinear geometry (NLGEOM) option of the Abaqus Explicit code is used in the FE model to reflect the deformation of the peened material (target). To simulate the shot–component interactions, the kinematic contact algorithm implemented in Abaqus is selected (Jebahi and Gakwaya [15]). Many researchers have noted that it is significative to account for shot–shot interactions (Tu and Delbergue [13]). Thus, collisions between shots are not still considered in this paper, consistent with the literature. For the reasons stated in the previous subsection, shots are modeled as identical rigid spheres. The Hertz–Mindlin nonslip contact law is employed to model the shot–shot interaction. A 3D random shot model considering the shot–shot interaction is shown in Figure 8a.

A quarter model (symmetry boundary conditions in the longitudinal and width directions) is employed to reduce the computation effort. The dimensions of the quarter Almen strip are 9.5 mm × 38 mm × 1.29 mm. To increase the accuracy of the simulation results, the strip is meshed with different element sizes in the depth direction. The type of element is the eight-node reduced integration element C3D8R. The mesh of the region that directly contacts shots is refined, the element size is 0.06 mm, the mesh size of the region far from the impact region is relatively large, and the total number of elements is 1,450,000. All displacements at the strip’s bottom are blocked, corresponding to the Almen holder of the test (an Almen strip held by four pretension bolts to the Almen holder), as shown in Figure 1. Figure 8b shows the improved DEM–FEM of a quarter Almen strip with normal impact corresponding to the experiment in Section 2.1.

There are several assumptions about the random multiple-shot model developed in this study: (1) all shots have spheres of the same radius and the same initial velocity; (2) collisions between shots are not considered; (3) the original surface of the target is assumed as smooth, regardless of the initial residual stress caused by machining and heat treatment. For a given shot type and impact angle, shot peening effects depend almost solely on the coverage and Almen intensity.

#### 3.4.2. The Second Model: Springback Implicit FEM for Removal Constraints

**Figure 9:**
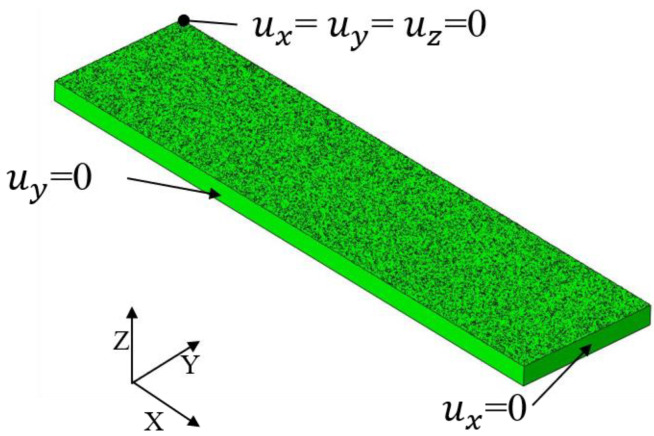
Springback implicit FEM for removal constraints.

The boundary conditions illustrated in Figure 9 are applied to simulate the removal constraints and prevent rigidity in the Springback simulation. Because it is a quarter model, the symmetry plane adopts the symmetry constraint.

## 4. Results

### 4.1. Almen Intensity and Arc Height

#### 4.1.1. Experimental Measures

Arc height values measured under different air pressures *P* and numbers of passes *W* during the experiment are shown in Table 3.

To determine the saturation points, the experimental data are as follows [6]:

(8) $$A_{h}=A_{1}\left[1-\exp(-W/B_{1})+C_{1}\right]$$

where *A_h_* is the measured arc height, *W* is the number of passes, and *A*_1_, *B*_1_, and *C*_1_ are fitting parameters.

**Figure 10:**
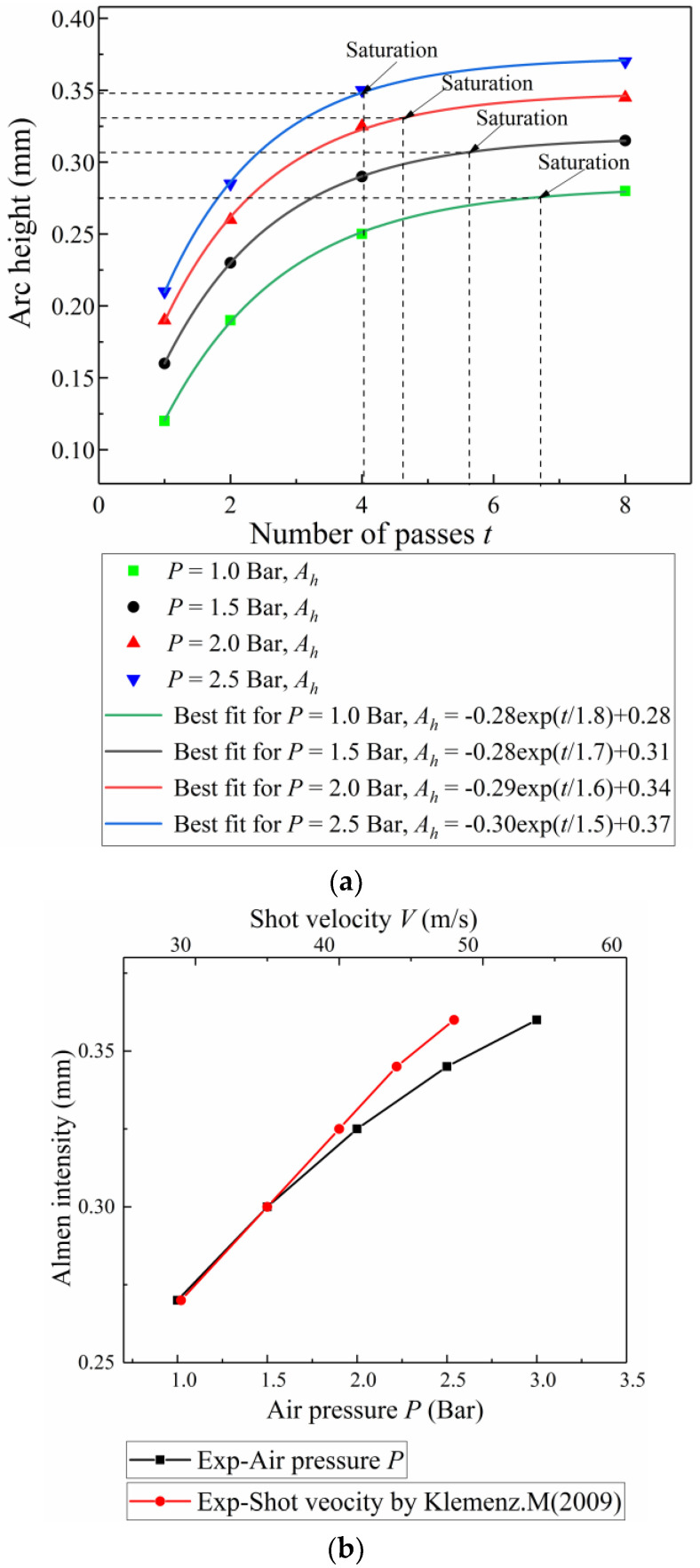
Almen intensity test. (**a**) Saturation curves of Almen strips with different air pressures for normal impact. (**b**) Relationship between the Almen intensity and air pressure, shot velocity by formula introduced by Klemenz. M (2009).

The number of passes at saturation, *W_S_*, is defined below:

(9) $$1.1A_{h}(W_{s})=A_{h}(2W_{s})$$

where $A_{h}(W_{s})$ is the Almen intensity. As shown in Figure 10a, the arc height increases with additional peening passes *W*; the Almen intensity varies approximately linearly with the air pressure, as shown in Figure 10b. Similar studies include Kumar and Sampath Kumaran [7] and Mohamed and Farhat [25].

To compare the experimental results with the simulation results, the relationship between air pressure and shot velocity was estimated by the semi-experiential formula introduced by Klemenz and Schulze [26], which is shown in Equation (10).

(10) $$V=\frac{163.5P}{1.53M+10P}+\frac{295P}{0.598d+10P}+48.3P$$

where *V*, *P*, *M*, and *d* represent the velocity of the shot ball (m/s), the air pressure (MPa), the mass flowrate (kg/min), and the diameter of shot balls (mm), respectively. For a given shot type (S230 steel shots with a diameter of 0.58 mm) and mass flowrate of 5.0 kg/min, the relationship between Almen intensity and shot velocity is shown in Figure 10b.

#### 4.1.2. Simulation Results

##### (1) Deformation Characteristics

**Figure 11:**
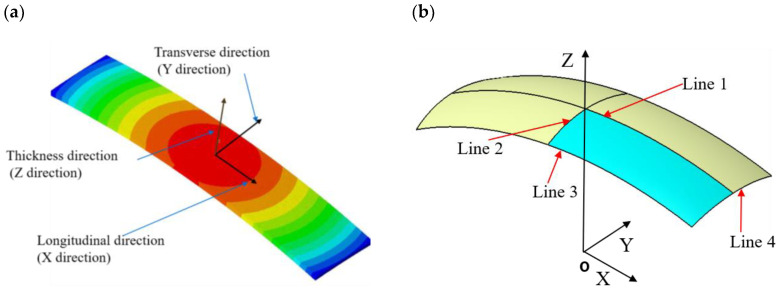
Shot peen forming. (**a**) Vertical deflection of a quarter Almen strip with a total arc height of 1.1 mm. (**b**) Four lines evaluated as the deflections along axes X and Y.

**Figure 12:**
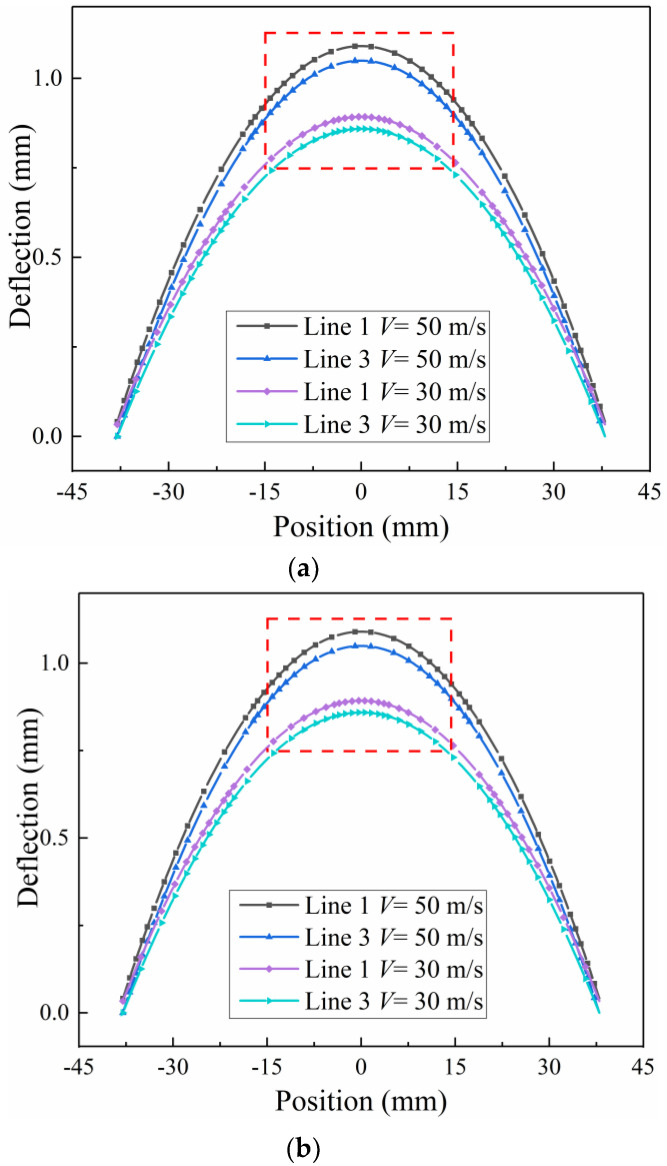
Coordinate system on the Almen strip and definition of the four lines. (**a**) Calculated deflections along the X or Y direction for lines 1, 2, 3 and 4. (**b**) The detailed view shows the arc height measurement.

Figure 11a shows that the compressive residual stress pattern after shot peening is the same in the longitudinal direction (along X) and transverse direction (along Y). The calculated deflections of curvature can then be related to the arc height. For illustration, the deformed Almen strip can be idealized as a spherical surface. Suppose that there are four lines evaluated as the deflections along axes X and Y, as illustrated in Figure 11b. Figure 12a shows four curves of the Almen strip with different shot velocities after shot peening. It is obvious that Line 1 and Line 2 almost completely coincide, indicating that a spherical surface is present in the middle of the strip. Figure 12b shows that the deflections obtained by Line 3 are less than those obtained by Line 1.

##### (2) Arc Height

**Figure 13:**
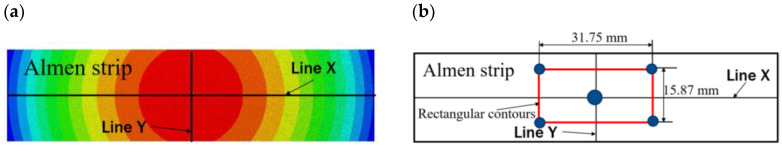
Direct measurement of arc height. (**a**) Deflection of the Almen strip. (**b**) Measured longitudinal deflection and transverse direction of the rectangular contours.

The arc height of the Almen strip can then be related to the deflection of lines X and Y, as shown in Figure 13a. In actual shot peening, the strips are subjected to the shot stream, and their free deflection over a 31.75 mm × 15.87 mm area is measured with an Almen gauge (see Figure 3). Thus, the longitudinal deflection and transverse direction of the rectangular contour area are used to calculate the arc height, as shown in Figure 13b.

The total arc height *A_h_* can be evaluated as the sum of arc heights from two curvatures, which is expressed by Equation (11):

(11) $$A_{h}=h_{l}+h_{t}$$

**Figure 14:**
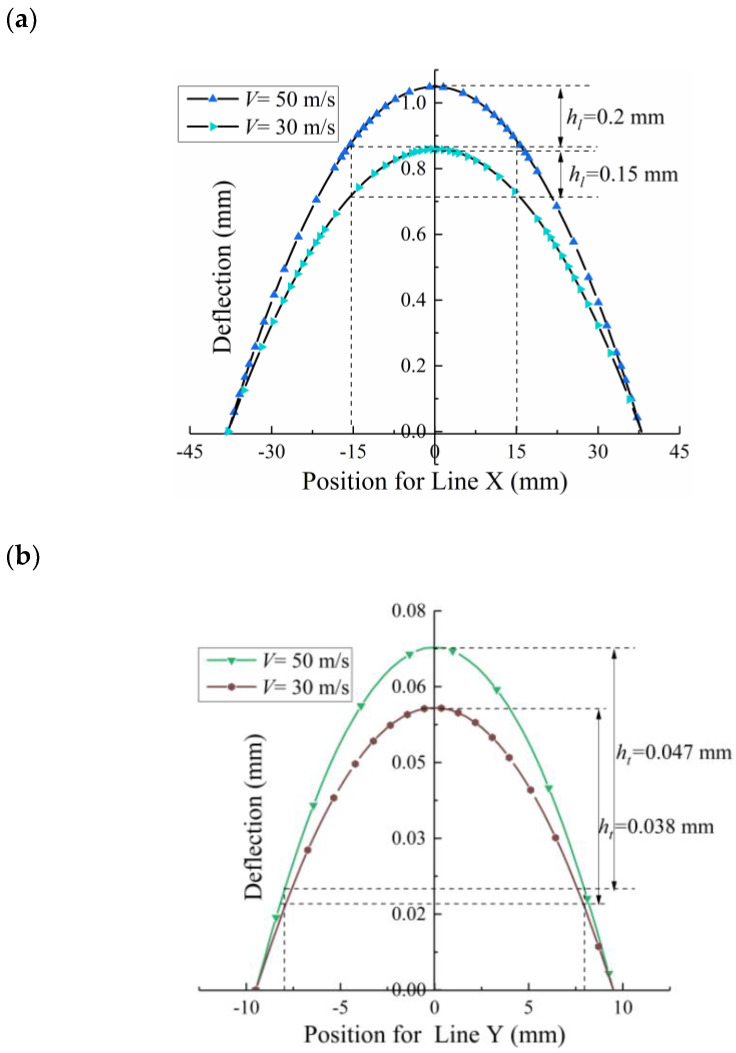
Total deflection. (**a**) Along the length profile and (**b**) across the width profile.

For example, when the shot velocity *V* is 30 and 50 m/s, the longitudinal arc height, *h_l_*, is approximately 0.15 and 0.2 mm, respectively, as shown in Figure 14a. The corresponding transverse arc height, *h_t_*, is approximately 0.038 and 0.047 mm, as shown in Figure 14b. Thus, it can be assumed that the compressive residual stress pattern is the same even in the transverse direction. Using Equation (3), the transverse arc height is approximately a quarter of the longitudinal arc height under the condition of the same radius of curvature *R*. In addition, the total arc height *A_h_* is as follows:

(12) $$A_{h}=h_{l}+h_{t}\approx \frac{5}{4}h_{l}$$

##### (3) Almen Intensity

**Figure 15:**
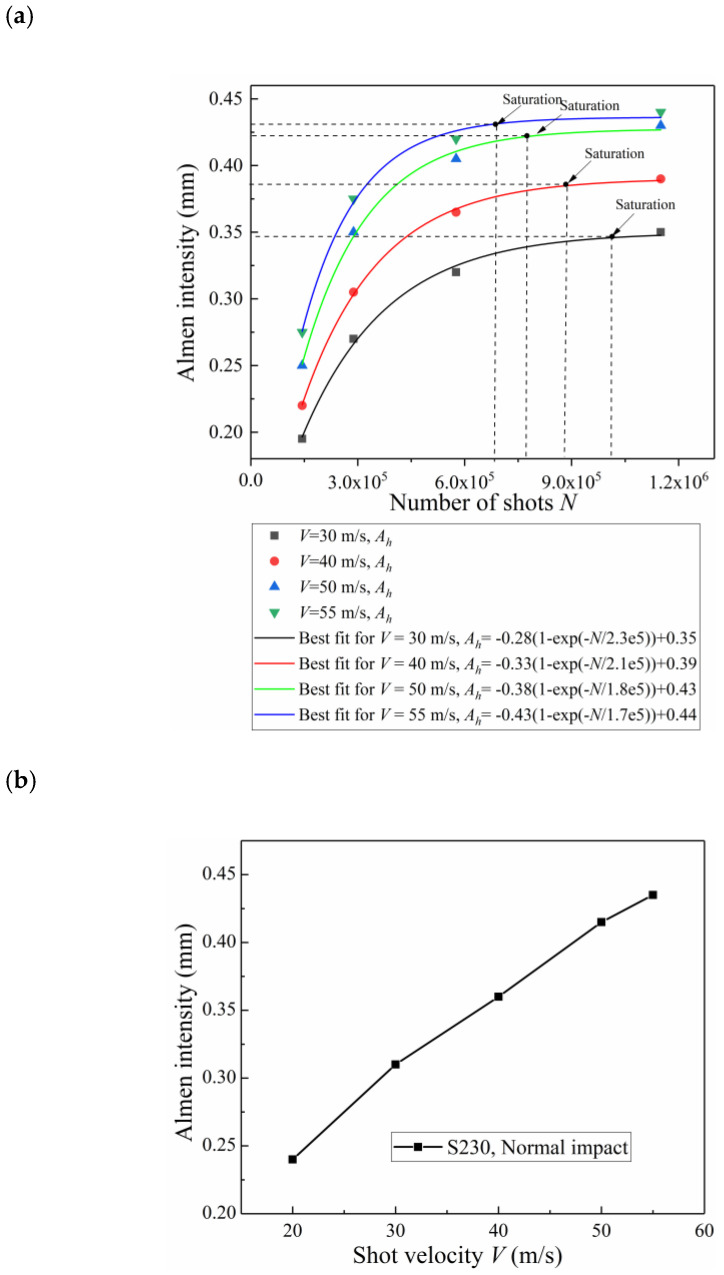
Simulation of the Almen intensity test. (**a**) Saturation curves of Almen strips with different shot velocities, corresponding to impingement angle *θ* = 90°. (**b**) The relationship between the Almen intensity and shot velocity.

The Almen intensity is defined as the arc height of the Almen strip at saturation point. To determine the saturation points, the simulation results are fitted as follows:

(13) $$A_{h}=A_{2}\left[1-\exp(-N/B_{2})+C_{2}\right]$$

where *A_h_* is the calculated arc height; *N* is the shot number; and *A*_2_, *B*_2_, and *C*_2_ are fitting parameters. The number of shots at saturation, *N_S_*, is defined below:

(14) $$1.1A_{h}(N_{s})=A_{h}(2N_{s})$$

where *A_h_(N_s_)* is the Almen intensity. Figure 15a presents the saturation curves of the Almen strip with different shot velocities, corresponding to impingement angles *α* = 90°. The Almen intensity values increase as the shot velocity is increased from 30 to 55 m/s. Figure 15b shows that the shot velocity presents an almost linear trend with the Almen intensity, which is consistent with the experimental results presented by Miao and Demers [6].

### 4.2. Validation

#### 4.2.1. Almen Intensity Validation

**Figure 16:**
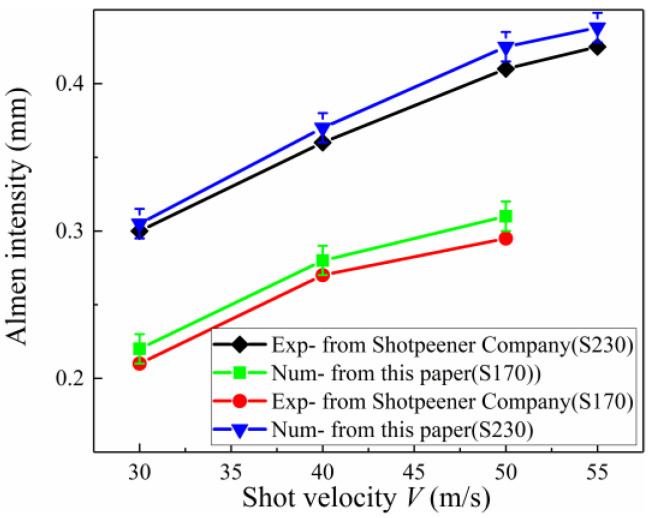
Numerical and experimental relationships between Almen intensity and shot velocity for two shot sizes and impingement angle *θ* = 90°.

In the previous sections, an improved DEM-FEM model of a large number of shots was developed to simulate the process of Almen intensity testing at minimal cost. Using this model, a simulation method was proposed to correctly predict the Almen intensity from an actual Almen intensity test in the shot peening industry. To validate this improved DEM-FEM and methodology, a comparison between the numerical and experimental results from Almen intensity curves in the literature [27] are given in Figure 16. The saturation curve and the saturation point obtained by the improved method in this paper and considering transverse deformation are obviously greater than those obtained by Jebahi and Gakwaya [15]. Furthermore, the Almen intensity curves obtained by the improved method in this paper are very close to those from the Shotpeener Company.

#### 4.2.2. Residual Stress Validation

**Figure 17:**
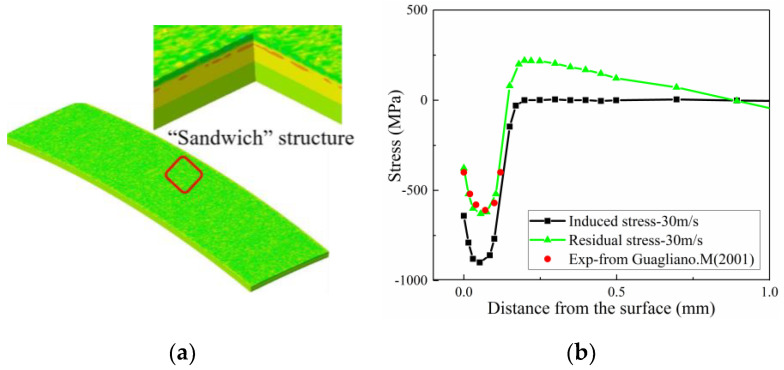
Removal constraints for the Almen strip. (**a**) “Sandwich” structure. (**b**) Comparison of the induced stress, residual stress from this paper and residual stress from Guagliano [16].

Residual stress develops from induced stress. The deformation of the Almen strip was simulated by modifying the boundary constraints. The Springback model of the real Almen strip was used to simulate removal constraints corresponding to removing the strip from the Almen holder in the shot peening experiment. After removing the constraint, the upper surface expands, the lower surface is compressed, and the middle is pulled to achieve balance. Figure 17a shows that the section of the strip is divided into the compressive stress zone of the surface layer, tensile stress zone of the middle layer, and compressive stress zone of the bottom layer after the release of constraints, such as a “sandwich” structure. Moreover, the stress on the Almen strip is released from the induced stress into residual stress. Figure 17b shows the stress along the thickness of the Almen strip before and after removal of the bolts. Guagliano [16] provided experimental data for SAE 1070 subjected to 0.3 mmA intensity due to S230 shots. The improved DEM-FEM simulations in the current study have been done with S230 shots to 30 m/s. It is shown in Figure 12b that when the residual stress profiles obtained by improved DEM-FEM are compared with ones that were experimentally measured, the agreement is satisfactory.

## 5. Discussion

The simulation method proposed in this paper further simulates the real Almen intensity test, which is not achieved by other traditional methods. The advantages are mainly reflected in the following aspects.

### 5.1. Comparison Study of Almen Intensity by Improved DEM-FEM Method and Other Existing Methods

**Figure 18:**
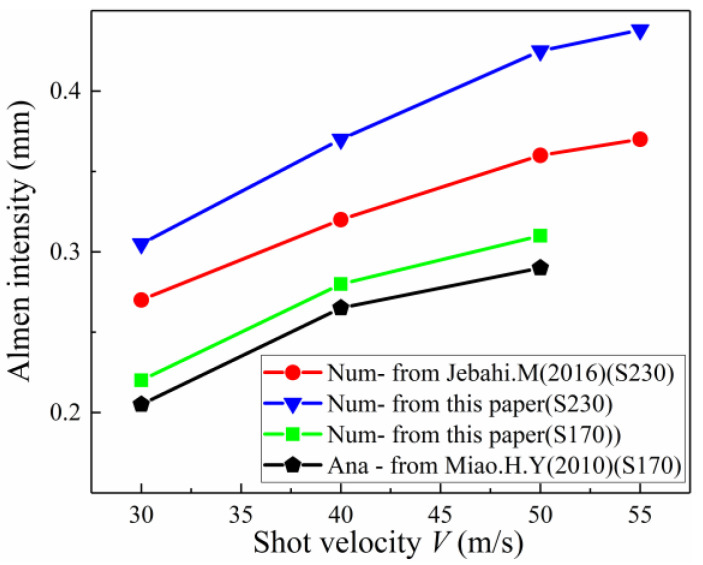
Comparison of Almen intensity for: the improved DEM-FEM in this paper, existing numerical method from Jebahi. M (2016), and analytical model method from Miao. H. Y (2010).

Figure 18 compares Almen intensity obtained by different methods as a function of shot velocity for two shot sizes: S170 steel shots (average diameter *D_ave_* = 0.504 mm), and S230 steel shots (*D_ave_* = 0.58 mm). Jebahi and Gakwaya [15] used a numerical method and elastic theory to calculate arc height and ignored the effect of transverse deformation by using Almen Type A strips and S230 steel shot. Miao and Larose [8] proposed an analytical model for predicting the Almen intensity and took the effect of transverse deformation into account by using Almen Type A strips and S170 steel shot.

**Figure 19:**
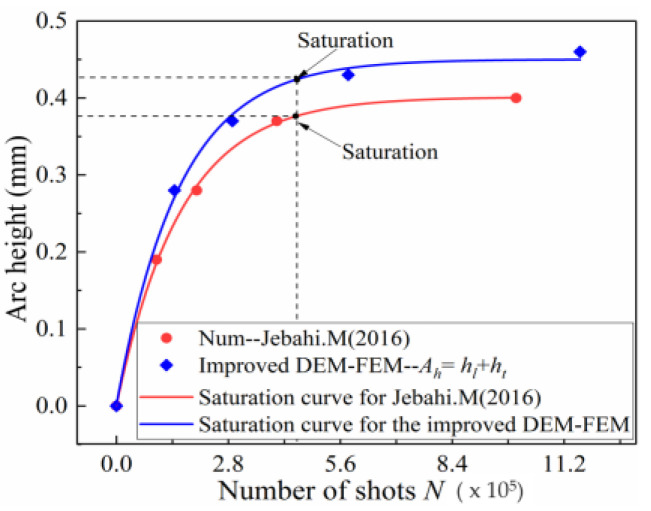
Influence of the transverse deformation on Almen intensity for shot velocity: 50 m/s and S230 steel shot.

**Figure 20:**
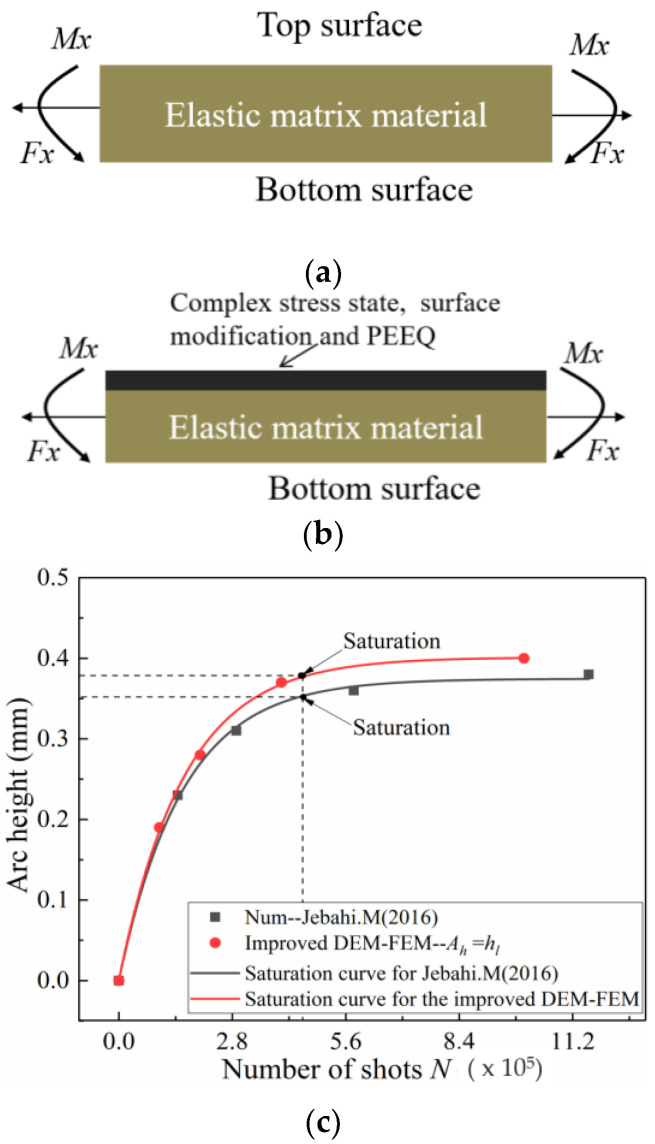
Influence of the plastic layer on deformation. (**a**) The elastic state used in the current equivalent method. (**b**) The plastic layer considered in the improved DEM-FEM. (**c**) Comparison of saturation curve for shot velocity: 50 m/s and S230 steel shot.

In both cases, Figure 18 shows that the improved DEM-FEM results for different shot velocities are slightly more than the analytical results of Miao and Larose [8], and obviously more than those obtained by JebahiIn addition Gakwaya [15]. Possible explanations for this phenomenon include:

(1) One of the main factors affecting the accuracy of arc height is transverse deformation. The transverse deformation of the strips, however, is often neglected when existing methods are used to obtain the arc height. For example, Jebahi and Gakwaya [15] used a numerical method and elastic theory to calculate arc height and ignored the effect of transverse deformation. Figure 19 shows that the saturation curve obtained by the improved method in this paper is obviously more than that obtained by Jebahi and Gakwaya [15]. Although transverse bending is smaller in the relative longitudinal direction than in other directions, its contribution to the total arc height is not small and should not be neglected. The transverse arc height is approximately one-fifth of the total arc height.
(2) Another of the main factors affecting the accuracy of arc height is the plastic layer. The real Almen strip solid element model is used instead of the representative unit, shell element and other equivalent models in this paper. The PEEQ, complex stress state, and surface morphology on the surface of the real Almen strip model are inherited by the Springback model. Moreover, for the existing method, the material model for calculating arc height is still the same elastic constitutive model as the material model before shot peening. However, the effect of surface hardening due to shot peening is not considered, as shown in Figure 20a. In fact, the residual stress, PEEQ and surface roughness on the surface of the real Almen strip are the mechanisms for improving the fatigue life after shot peening, as shown in Figure 20b. Ongtrakulkij and Khantachawana [28] reported that shot peening is a surface modification that can increase the bending strength by increasing the hardness and residual compressive stress. Bhuvaraghan and Srinivasan [4] proposed that the unit cell approach without superposition of the elastic theory is likely to provide accurate results when the thickness is high compared to the thickness of the plastic layer. It can be concluded that the surface morphology, complex stress state, and PEEQ of the plastic hardening layer influence the deformation of the strip. The saturation point obtained by the improved method in this paper and ignoring transverse deformation is slightly more than that obtained by Jebahi, Gakwaya et al. (2016), as shown in Figure 20c. This difference may be explained by the fact that PEEQ, the complex stress state and surface morphology of the plastic hardening layer decrease the bending stiffness of the strip. The influence of the plastic layer on deformation is about 10%. Currently, it is clear how the plastic hardening layer affects the arc height, and this factor should not be ignored. The proposed improved DEM-FEM approach can avoid this problem by considering the surface morphology, complex stress state, and PEEQ naturally.

### 5.2. Comparison Study of Arc Height by Improved DEM-FEM Method and Existing Shell Element Method

**Figure 21:**
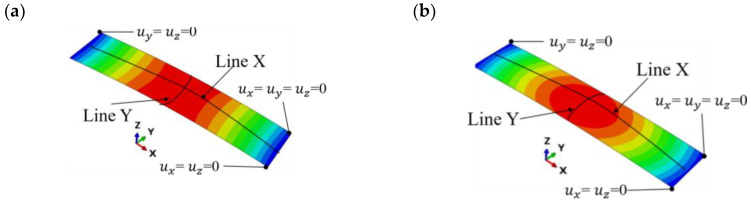
(**a**) Saddle shape by induced stress $\sigma_{xx}$. (**b**) Spherical shape by induced stress $\sigma_{xx}$ and $\sigma_{yy}$.

We now compare the real Almen strip model in this paper with the existing shell element method. The shell element method strategy presented by Gariépy and Larose [29] is applied to simulate peen forming operations on the Almen strip for comparison. When transverse deformation is not considered, the induced stress state in the longitudinal direction ($\sigma_{xx}$) is often selected as the input data to incrementally establish the deformed shapes, and the strip is in the shape of a saddle, as shown in Figure 21a. When transverse deformation is considered, the induced stress state in both directions ($\sigma_{xx}$ and $\sigma_{yy}$) is selected as the input data to incrementally establish the deformed shapes, and the strip is spherical, as shown in Figure 21b.

**Figure 22:**
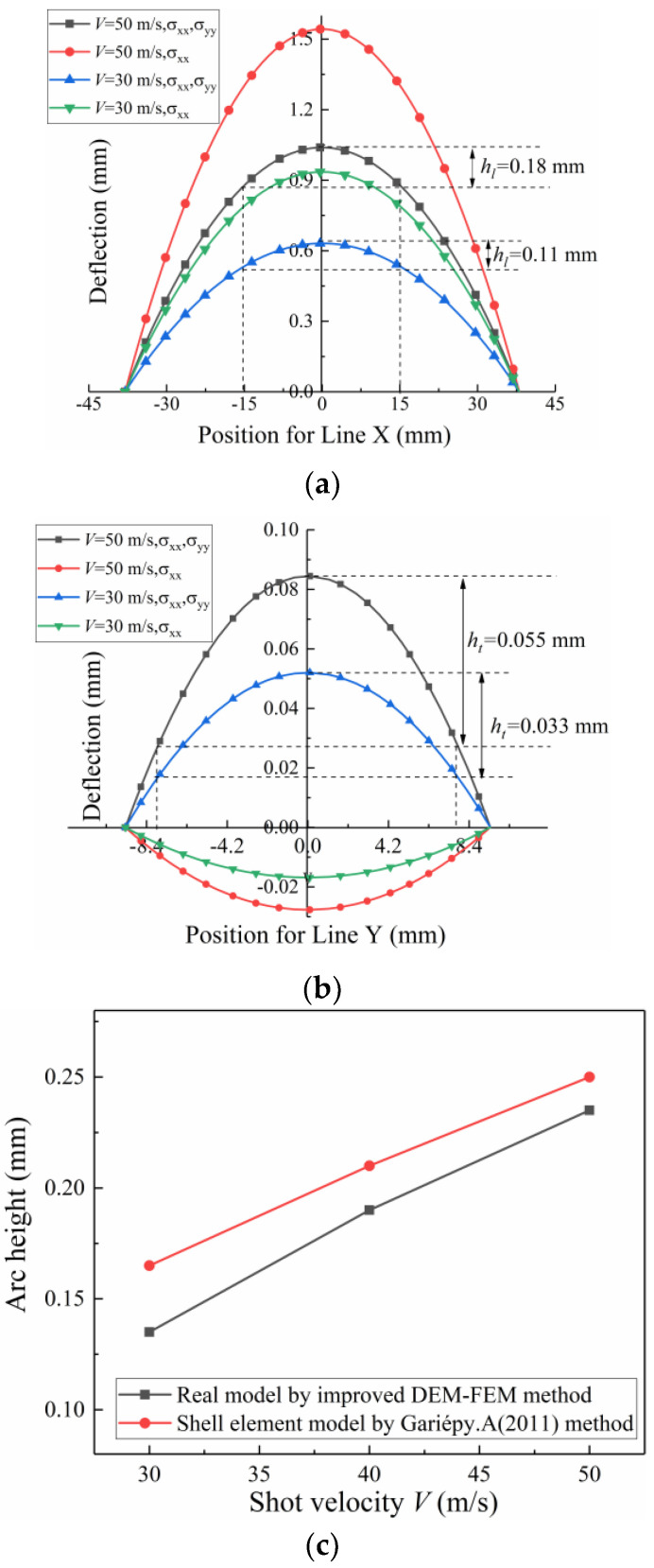
Current method. (**a**) Along the length profile and (**b**) across the width profile. (**c**) Comparison of arc height for the improved DEM-FEM method and existing shell element method.

Figure 21 shows that the induced stresses in different directions selected as the input data have a great influence on the deformation of the strip. Figure 11a and Figure 21b show that the deformation pattern (spherical shape) obtained by the shell element model is consistent with that obtained by the direct simulation method when the induced stress in both directions ($\sigma_{xx}$ and $\sigma_{yy}$) is considered. Analogously, when the shot velocity *V* is 30 and 50 m/s, the longitudinal arc height, $h_{l}$, is approximately 0.11 and 0.18 mm, respectively, as shown in Figure 22a. The corresponding transverse arc height, $h_{l}$, is approximately 0.033 and 0.055 mm, as shown in Figure 22b. Figure 22c shows that the arc height obtained by the existing method based on shell element is less than that obtained by the method proposed in this paper. Possible explanations for this reason include: (1) part of the induced stress information of the node is lost. Since the stress along each line of the representative volume in the thickness direction is different, the average induced stress profiles along the thickness direction of the representative unit are extracted and added to the entire Almen strip shell model to calculate the deformation indirectly in this current simulation process, which is different from the real situation. For example, the induced stress state in the longitudinal direction ($\sigma_{zz}$) is often not considered. The direct simulation method proposed in this paper avoids information loss and distortion by spring back model. (2) Compared with the case of the shell element method, the whole line is constrained (see Figure 21). The constraint on one point (see Figure 9) in the Springback model of the real Almen strip is closer to the details of the real experiment.

## 6. Conclusions

The present study aimed to replicate the real shot peening process as closely as possible and replace the existing method to calculate the precise deformation of shot peened strips. This paper attempted to reveal the new phenomenon generated by the real and large number of shots impacting a real-size Almen strip. First, the main numerical models and equivalent methods were briefly reviewed and discussed to identify their major advantages and limitations. To overcome some of these limitations and further understand the shot peening process, an improved DEM-FEM method for the Almen intensity test by a real and large number of shots was proposed. The following conclusions can be drawn from the results of these simulations and experiments:

(1) Direct simulation of the Almen intensity test by a real and large number of shots is proposed and then implemented for the first time. The deformation of the Almen strip is modeled using the springback implicit FEM to accurately model the mechanical response in terms of deformation. The arc height of the Almen strip is directly obtained after the removal of the bottom constraint, rather than by the equivalent method. Therefore, the method proposed in this paper is closer to the real shot peening process. Though the computational effort involved in this simulation may be higher than the traditional method, it is now possible to obtain the Almen intensity without using any post-analysis corrections.
(2) The existing simulation method is mainly to calculate the number of shots by means of coverage rate, but this method cannot reflect the process parameters in the actual shot peening process. A new method for calculating the number of shots needed for simulation based on experiments and process parameters such as the mass flowrate, nozzle movement speed and nozzle–workpiece distance is presented in this paper, and a formula for calculating the number of shots is derived. This new method is used to calculate the number of shots and then to link the shot peening experiment and simulation.
(3) The simulation results and experimental results show that the transverse deformation cannot be ignored when calculating the arc height; the transverse arc height is approximately one-fifth of the total arc height. The influence of the surface plastic layer on the deformation of the Almen strip should be considered; the influence of the plastic layer on deformation is about 10%. The method presented in this paper naturally takes the transverse deformation and surface plastic layer into account. Thus, the Almen intensity calculated by the direct simulation method therefore is in better agreement with the experimental results and literature data than that calculated by existing methods.

Finally, the improved DEM-FEM method proposed in this paper, which can obtain the Almen intensity as a function of shot velocity, and further accurately predict the deformation, presents a major step towards quantitative precise shot peening forming technology.

## Figures and Tables

**Table 1:** Mechanical properties and Johnson–Cook constants of the Almen strip (taken from Jebahi and Gakwaya [15]).

Mechanical Properties				Johnson–Cook Constants				
Material	E (GPa)	ν	ρ (kg/m^3^)	A (MPa)	B (MPa)	C	n	m
Shot (Stainless steel)	210	0.3	7850					
Almen strip (SAE 1070)	205	0.29	7800	1048	600.8	0.0134	0.234	0

**Table 2:** The relationship between the shot number *N* and peening passes *W* for the containing shot velocity.

Case	*V_R_* (mm/min)	*T*	*N*
Strip 1	1000	1	144,000
Strip 2	1000	2	288,000
Strip 3	1000	4	576,000
Strip 4	1000	8	1,150,000

**Table 3:** Arc height (mm) under different air pressures *P* (bar) and numbers of passes *W*.

*W*	*P*			
	1.0	1.5	2.0	2.5
1	0.11	0.13	0.17	0.23
2	0.15	0.18	0.22	0.27
4	0.19	0.22	0.26	0.32
8	0.20	0.24	0.28	0.33
